# Validation of AIDS-related mortality in Botswana

**DOI:** 10.1186/1758-2652-12-24

**Published:** 2009-10-24

**Authors:** Negussie Taffa, Julie C Will, Stephane Bodika, Laura Packel, Diemo Motlapele, Ellen Stein, Thierry H Roels, Gail Kennedy, El-Halabi Shenaaz

**Affiliations:** 1 BOTUSA (Botswana-USA), Centers for Disease Control and Prevention, Gaborone, Botswana; 2 Division for Heart Disease and Stroke Prevention, Centers for Disease Control and Prevention, Atlanta, Georgia, USA; 3 Department of Epidemiology & Institute for Global Health, University of California, San Francisco, California, USA; 4 Department of Policy, Planning, Monitoring & Evaluation, Ministry of Health, Gaborone, Botswana; 5 Department of Primary Health Care, Ministry of Health, Gaborone, Botswana

## Abstract

**Background:**

Mortality data are used to conduct disease surveillance, describe health status and inform planning processes for health service provision and resource allocation. In many countries, HIV- and AIDS-related deaths are believed to be under-reported in government statistics.

**Methods:**

To estimate the extent of under-reporting of HIV- and AIDS-related deaths in Botswana, we conducted a retrospective study of a sample of deaths reported in the government vital registration database from eight hospitals, where more than 40% of deaths in the country in 2005 occurred. We used the consensus of three physicians conducting independent reviews of medical records as the gold standard comparison. We examined the sensitivity, specificity and other validity statistics.

**Results:**

Of the 5276 deaths registered in the eight hospitals, 29% were HIV- and AIDS-related. The percentage of HIV- and AIDS-related deaths confirmed by physician consensus (positive predictive value) was 95.4%; however, the percentage of non-HIV- and non-AIDS-related deaths confirmed (negative predictive value) was only 69.1%. The sensitivity and specificity of the vital registration system was 55.7% and 97.3%, respectively. After correcting for misclassification, the percentage of HIV- and AIDS--related deaths was estimated to be in the range of 48.8% to 54.4%, depending on the definition.

**Conclusion:**

Improvements in hospitals and within government offices are necessary to strengthen the vital registration system. These should include such strategies as training physicians and coders in accurate reporting and recording of death statistics, implementing continuous quality assurance methods, and working with the government to underscore the importance of using mortality statistics in future evidence-based planning.

## Background

Accurate and standardized systems for the reporting of causes of death are essential in order to monitor the impact of public health interventions and analyze mortality trends over time [1,2]. Although low-income and middle-income countries recognize the importance of timely and accurate health statistics, the death registration systems in these settings are frequently inadequate due to incomplete and delayed reporting of deaths, missing data, inaccurate reporting of the cause of death, and incorrect coding of underlying and contributory causes of death [2-4].

Deaths specifically due to HIV and AIDS are under-reported in low- and middle-income countries [2]. As a result, few countries have mortality data systems that are adequate to shape public health policy and programmes [2]. Various societal and legal factors may complicate the reporting of deaths from HIV and AIDS [3,5]. Lack of reporting of deaths outside health institutions, physician failure to report a death, the social stigma of HIV [5], missing HIV-specific documentation in the medical record, and lack of a clear primary and/or contributory causes of death are the commonly cited reasons [6].

Even when HIV and AIDS is listed as a cause of death on a death certificate, inaccurate coding of the death may occur [6]. In South Africa, it has been suggested that additional training for health care professionals in proper completion of death certificates may improve mortality reporting [6].

Botswana is believed to have one of the better vital registration systems in Africa. In 2004, 91% of all deaths occurred in a hospital [7], and this increased to 96% in 2005 [personal communication, Botswana Central Statistics Office, 2008]. In-hospital deaths are well captured in the national vital registration system. Nonetheless, it is likely that HIV and AIDS as causes of death are under-reported; the health statistics report for 2004 reported that HIV and AIDS deaths accounted for 19.8% of all deaths [7], which is a substantially smaller percentage than found in more recent years.

Botswana is the first African country to provide free antiretroviral (ARV) treatment for its citizens, starting in 2002. By the end of 2007, more than 80% of those eligible had received treatment. Much reduction in the impact of HIV and AIDS deaths is anticipated, but it is not known whether the current vital registration system will be able to accurately capture this reduction. To evaluate the vital registration system for this purpose, we focus on three major objectives: (1) assessing the validity of HIV and AIDS deaths reported from hospitals; (2) characterizing the extent to which deaths related to HIV and AIDS fail to be recorded as such; and (3) providing statistics on HIV- and AIDS-related deaths adjusted for under-reporting.

## Methods

### Design and sample

We conducted a retrospective study to validate cause-of-death reporting among a sample of deaths occurring in selected hospitals in 2005. We restricted our analysis to deaths occurring in hospitals because almost all deaths in Botswana occur and are verified there [7]. We used a convenience sample of eight from the 32 hospitals (excluding one military and one private hospital) located throughout the country. We chose the two referral hospitals located in the two largest cities in Botswana, and then six additional hospitals that were spread throughout the country, representing areas with different geographic characteristics and population densities.

Using the vital registration database, deaths were stratified into HIV- and AIDS-related or non-HIV and AIDS-related. Systematic samples of 10% of HIV- and AIDS-related deaths and 50% of non-HIV- and AIDS-related deaths were planned for study. We chose 50% as the sampling percentage for non-HIV- and AIDS-related deaths because we were primarily interested in under-reporting of these deaths and wanted to ensure adequate power to provide precise validity estimates.

Over-reporting among HIV- and AIDS-related deaths was a secondary research question and we believed that the degree of misclassification would be less; thus, we chose the smaller sampling percentage of 10%. These sampling percentages were not determined using formal power calculations. Sampling was achieved after sorting all deaths by the two disease categories and by hospital record number. Every other record was selected from among non-HIV- and AIDS-related deaths and every 10^th ^record was selected from among HIV- and AIDS-related deaths. The cause of death as recorded in the government database (see the process described below) was then compared to cause of death as reported by physician consensus after detailed chart reviews.

### Government mortality statistics

For each death in the hospital, an MH 017 form [see Additional file 1] is completed by a physician. The MH 017 includes the physician's assessment of the following: (1) the underlying cause of death; (2) the immediate or direct cause of death; (3) up to two antecedent causes; and (4) contributing conditions to death that were not related to the cause. The form is then filed with the Health Statistics Unit at the Ministry of Health.

Government employees who are trained to use the Tenth International Classification of Diseases (ICD-10) codes and algorithms [8] enter the information into a government database. If the ICD-10 codes of B20-B24, HIV diseases, are recorded as an underlying, immediate or antecedent cause in Section I of the mortality cause-of-death portion of the MH 017, then the death is officially recorded as an HIV- and AIDS-related death. Although rare [personal communication, Ministry of Health, 2008], sometimes this portion is not completed at all. However, the discharge status from the hospital is recorded as a death, and HIV- and AIDS- related is recorded as the diagnosis in the morbidity portion of the form. When this happens, the death is also recorded as an HIV- and AIDS-related death.

### Gold standard comparison (Method A)

Three study physicians and three study nurses practicing in Botswana reviewed hospital medical records of the selected decedents. The physicians were trained as general practitioners and/or family medicine doctors. The nurses were trained as family nurse practitioners. They normally treat patients alongside the doctors, in both hospitals and clinics. The study physicians and nurses were unaware of the causes of death recorded in the government database.

Physicians and nurses received training on proper review of the medical record and the use of the data abstraction form. The data abstraction form collected information regarding the most recent admission, patient demographics, diagnosis from previous admissions at that hospital, history and results of HIV tests, referrals for HIV care or ARV therapy, use of cotrimazole and other medications suggestive of HIV infection, HIV-related laboratory tests (CD4, viral load), WHO HIV clinical staging 4, and for children, information regarding their mothers' use of the prevention of mother to child transmission programme.

The data abstraction form also provided us with a sense of the completeness of information in the charts. For example, we found that HIV-positive status was unknown for 67.2% of decedents. Pilot testing of the abstract form was done at a hospital that was not included in the study and was completed by the study physicians and nurses. Revisions were made to address the issues uncovered during the pilot test.

Each physician-nurse team (one physician and one nurse) independently reviewed the medical charts and abstract form, and determined the primary (i.e., immediate or direct) and contributory causes of death (Table 1). Once this was done, the three lead physicians discussed each decedent's medical record and arrived at the consensus causes of death (up to three contributory and one primary cause).

**Table 1:** Form used to gather physicians' estimates of primary and contributing causes of death

	Reviewer 1	Reviewer 2	Reviewer 3	Consensus
Primary COD:				

Contributing cause A:				

Contributing cause B:				

Contributing cause C:				

The contributory causes of death were used in the discussions by the three lead physicians, especially if they disagreed about the primary or underlying cause of death. One of the most challenging aspects of developing a consensus on primary cause of death is determining the sequence of events leading to the death. Listing contributory causes of death allows each physician to understand how the other physicians viewed the sequence of events leading to the death, thus facilitating the process of consensus development.

The data abstraction forms were then submitted to the Health Statistics Unit, and the government coder inserted the consensus primary cause of death as HIV and AIDS related or not using the ICD-10 coding system. For example, in the case of HIV/AIDS leading to tuberculosis (TB), the underlying cause would be HIV/AIDS, and TB would be listed as the secondary cause of death. The ICD-10 code used was B20.0.

However, if the sequence of events was not clear, then a combination code was used that allowed classification of two diagnoses, or a diagnosis with an associated sign or symptom, or a diagnosis with an associated complication. The consensus codes were primarily used for this determination, but in those situations in which a consensus code was missing (<1% of deaths), the coder used the causes of death listed by the individual physicians and the ICD-10 algorithms to make the determination.

### Alternative method and definitions of HIV- and AIDS-related deaths

We used a number of definitions for HIV- and AIDS-related deaths, which allowed us to develop a range of values to estimate the percent misclassified. This is useful given the various ways that HIV- and AIDS-related deaths have been previously defined. For example, some studies that have examined misclassification have used the conditions listed in Appendix 1 to determine whether a death is HIV and AIDS related [9-11]. We have also used this list to produce alternative definitions, which we have labeled Methods B, C, and D. See Appendix 1 for these definitions.

### HIV- and AIDS-related mortality prevalence estimates

We used the proportion of HIV- and AIDS-related deaths, as determined by consensus of the three physicians, as the "true" mortality prevalence. We also examined how this would change if we used the experts' (three physicians employed by the Centers for Disease Control and Prevention and who are experts in HIV/AIDS) review, with varying definitions for HIV- and AIDS-related deaths (i.e., Methods B, C, and D).

### Data analysis

We originally targeted 364 of 1530 (10% of HIV- and AIDS-related deaths and a 10% contingency sample of the sampled charts) HIV- and AIDS-related deaths, and 1873 of 3746 (50%) non-HIV- and AIDS-related deaths for our validation study.

We found a total of 1827 of the 2237 (81.7%) charts to compare against the results of the government reporting process. This resulted in a 42% sample of non-HIV and AIDS deaths and a 17% sample of HIV- and AIDS-related deaths. Major reasons for missing charts included problems with matching the identifier found on the MH 017 with the identifier on the medical record, charts that were checked out and not returned, and temporary misplacement of charts.

We used the SAS statistical package, Surveyfreq procedure [12], to calculate validity statistics, including sensitivity, specificity, post-test probability given a negative test, positive predictive value, and negative predictive value. We weighted the sample units to obtain population estimates for the eight hospitals in aggregate. We used the proportion of deaths recorded as negative on the MH-017 that were determined to be positive by physician consensus as the measure of under-reporting of HIV- and AIDS-related deaths. We calculated the 95% confidence intervals for weighted percentages. We also examined under-reporting by age, gender and hospital to determine whether it varied by sub-population and/or facility.

## Results

**Figure 1:**
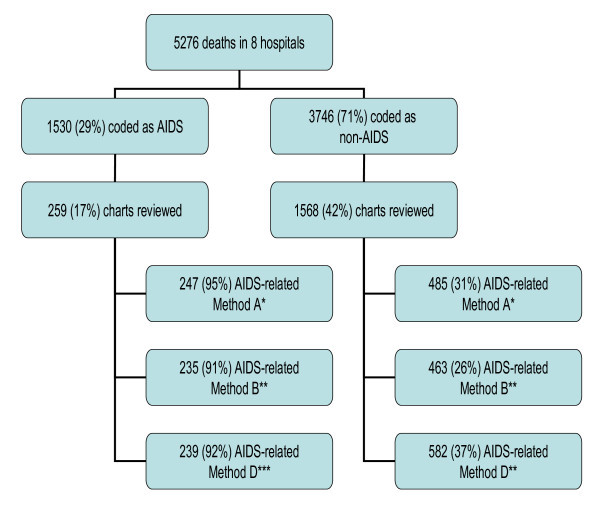
**Overview of sampling and results of chart reviews, eight hospitals, Botswana, 2005**. Note: The following legend show be used in interpreting the figure: * Physician consensus, ** Expert review using definitive definition, *** Expert review using definitive, probable, and possible definition.

In 2005, there were 11,949 total deaths in Botswana. Forty-four percent (5276) of these occurred in the eight study hospitals. Of these, 3746 (71%) were coded as not being HIV- and AIDS-related, and the remaining 1530 (29%) were coded as HIV- and AIDS-related (Figure 1).

In the study sample, of the 259 HIV- and AIDS-related deaths reported to the MOH, 247 were determined by Method A to be HIV- and AIDS-related by physician consensus (Table 2). In other words, the positive predictive value of the vital registration system is 95.5%. Of the 1568 non-HIV- and AIDS-related deaths in our sample, 1083 were determined to be non-HIV- and AIDS-related by physician consensus. Thus, the negative predictive value of the registration system is 69.1%.

**Table 2:** Misclassification of deaths reported to the Botswana Government, Method A, eight hospitals, 2005

			HIV- and AIDS-related death as determined by physician consensus		
			**Yes**	**No**	**Total**

HIV- and AIDS-related death as recorded in the vital registration database	Yes	Unweighted	247	12	259
		Weighted	1459 (*True Positives*)	71 (*False Positives*)	1530
	
	No	Unweighted	485	1083	1568
		Weighted	1159 (*False Negatives*)	2587 (*True Negatives*)	3746
	
	Total	Unweighted	732	1095	1827
		Weighted	2618	2658	5276

Weighted numbers are provided to adjust for the two different sampling fractions employed in this study. Weighting allows for accurate calculations of statistics such as the specificity and sensitivity of the vital registration database. See the Methods section for more details on sampling fractions.

The likelihood of a person having died from an HIV- and AID-related cause when the vital registration system indicated that they did not is 30.9% (i.e. under-reporting of HIV- and AIDS-related deaths or the post-test probability given negative recording in the vital registration system). The sensitivity of the government mortality reporting system in picking up HIV- and AIDS-related deaths is 55.7%. The specificity of the system in ruling out HIV- and AIDS-related deaths is 97.3%.

Government statistics indicate that 29.0% of deaths in 2005 were HIV and AIDS related. However, the physician consensus data indicates that the true percentage was 49.6%.

Under-reporting of HIV- and AIDS-related deaths in the vital registration system was lowest among decedents aged 50 and older (12.4%) and equivalent among male and female decedents (31%) [data not shown]. It also varied by hospital, ranging from 19.1% to 55.7%. The hospital that had 55.7% under-reporting was clearly an outlier, being significantly statistically different from all other hospitals.

Overall, using Method A (physician consensus), we found under-reporting of HIV- and AIDS-related deaths to be 30.9% (Table 3). Using an alternative method for validating the mortality reporting system (i.e., expert review), and using the most conservative definition of an HIV- and AIDS-related death (Method B, "definitive HIV/AIDS"); we found under-reporting of 29.5%. However, using a less restrictive definition of "definitive and probable" (Method C), we found 30.5% under-reporting. Finally, using the least restrictive definition (Method D, "definitive, probable, and possible"), we found 37.1% under-reporting.

**Table 3:** Validity statistics using four methods for determining an HIV- and AIDS-related death, Botswana Vital Registration System, 2005

Gold standard definition	Post-test probability given negative test^a^	Sensitivity (95% CI)	Specificity (95% CI)	Positive predictive value (95% CI)	Negative predictive value (95% CI)	"True" prevalence
Method A: physician consensus	30.9 (29.2-32.7)	55.7 (54.2-57.3)	97.3 (96.0-98.7)	95.4 (93.0-97.7)	69.1 (67.3-70.8)	49.6 (48.2-51.0)
Method B: experts review^b^	29.5 (27.8-31.3)	55.7 (54.0-57.3)	94.9 (93.2-96.6)	90.7 (87.5-94.0)	70.5 (68.8-72.2)	47.3 (45.7-48.8)
Method C: experts review^c^	30.5 (28.8-32.3)	54.8 (53.2-56.5)	94.8 (93.1-96.5)	90.7 (87.5-94.0)	69.5 (67.7-71.2)	48.0 (46.5-49.6)

Method D: experts review^d^	37.1 (35.3-38.9)	50.4 (48.9-51.9)	95.2 (93.5-97.0)	92.3 (89.3-95.2)	62.9 (61.1-64.7)	53.1 (51.6-54.7)

^a^Also known as under-reporting of HIV- and AIDS-related deaths by the vital registration system

^b^Using definite definition of HIV- and AIDS-related deaths

^c^Using definite and probable definition of HIV- and AIDS-related deaths

^d^Using definite, probable, and possible definition of HIV- and AIDS-related deaths.

Consequently, the "true" percentage of HIV- and AIDS-related deaths in the eight hospitals that we studied ranged from 48.8% to 54.4% (Table 3), depending on the method of validation and the definition of an HIV- and AIDS-related death.

We examined common causes of death other than HIV and AIDS (data not shown). The most common non-infectious causes of death listed among those coded as non-HIV- and AIDS-related deaths prior to the medical record review were hypertension, stroke, congestive cardiac failure, renal failure, trauma, hepatic failure, diabetes and cancer of the esophagus. It is important to note that as several causes of death were listed for each patient, this does not represent a mutually exclusive, ranked list of the most common cause of death.

## Discussion

In 2005, mortality statistics in Botswana indicate that approximately 29% of deaths were related to HIV and AIDS. In our validation study, we found that approximately half of all deaths we studied were HIV- and AIDS-related. This was true using a variety of validation methods.

We found the vital registration system to be 56% sensitive in reporting HIV- and AIDS-related deaths and 97% specific in ruling out HIV and AIDS deaths. Given that we found under-reporting of HIV- and AIDS-related deaths in eight of 32 public, missionary and mine hospitals, we caution the use of government mortality statistics to estimate the burden of HIV and AIDS in Botswana without the use of corrections for under-reporting.

Errors in government mortality statistics have been studied throughout the world [2] and in some African countries [6,9-11,13], and have been found to be widespread. As a result, many countries are examining their mortality statistics more closely with an eye toward improvement [6,9-11,13]. Some scientists have discouraged comparing the degree of under-reporting across countries or the true rate of HIV- and AIDS-related mortality due to varying validation methods across countries and samples that are non-representative of national deaths [9].

Even with similar validation methods, different patterns of cause-specific mortality have been shown to influence sensitivity and specificity rates [13]. It has been shown, for example, that verbal autopsy validation studies in Tanzania, Ethiopia, and Ghana have yielded sensitivity rates for TB and AIDS ranging from 56% to 82% and specificity rates ranging from 89% to 99.5% [13].

In the United States, death certificates listing diabetes anywhere on the certificate have been found to be 34.7% sensitive and 98.1% specific [14]. Clearly, cause-of-death statistics need improvement not only in Africa, but in other continents as well.

There are a variety of factors that are likely to have contributed to the under-reporting of HIV- and AIDS-related deaths in Botswana. First, it is important to note that hospital facilities in our study differed substantially in the degree of under-reporting. This implies that it is possible to improve statistics by examining the best practices in the hospitals with the lowest under-reporting and implementing them in the other hospitals. Other factors include HIV-associated stigma, which continues to be highly prevalent [15].

The need for death reports for burial may cause families to request that HIV not be listed as a cause of death, or physicians may assume that this is what families would wish. Physicians whose primary aim is to care for patients and their families may not fully appreciate the value in accurate and timely death statistics. Also, the limited number of government nosologists may have made it difficult for them to follow up with physicians to ensure that MH 017 forms are fully and accurately completed. There appears to be no current method for ensuring quality of reporting and, in the past, there has been little systematic training for physicians on completing the MH 017 and using the most recent versions of ICD coding.

Our study has a number of important strengths. First, we examined multiple geographic locations and samples from all deaths that occurred in eight hospitals. This allows us to generalize to those settings where almost half of all deaths occurred. Second, our method of validating mortality statistics employed additional information beyond re-review of death reports. Finally, we used various definitions of HIV- and AIDS-related deaths, which allowed us to estimate the degree of misclassification due to the definition itself. Our different methods yielded comparable results.

Despite these strengths, our study also has limitations. Information in the medical records may not have been complete, making it sometimes difficult for the study physicians to reach consensus on the causes of death. In addition, the physicians did not have the benefit of being present in the hospital before or at the time of death, which would have strengthened their ability to make the most accurate diagnosis. It is also possible that the study physicians overestimated the number of HIV- and AIDS-related deaths due to their awareness of the study objectives.

In the smaller hospitals, our sampling fraction of 10% for HIV- and AIDS-related deaths, even with contingency sampling, resulted in small sample sizes restricting our ability to study the performance of individual doctors. Finally, our best estimate for out-of-hospital deaths is 8.7% in 2004 [7] and 4.2% in 2005 [personal communication, Botswana Central Statistics Office, 2008].

Our results are only generalizable to the decedents from the areas served by the eight hospitals to the degree to which out-of-hospital deaths are proportionately few. To confirm these percentages, more research is needed on the number of deaths that occur outside the hospitals among families who do not need death certificates and are without insurance, and are unlikely to report a death to local officials.

With the broad use of antiretroviral therapy in Botswana, people with HIV and AIDS are likely to be living longer, as has been found in Brazil [16]. Given this, people with HIV and AIDS are more likely to die from chronic diseases, such as heart disease and diabetes [16]. Therefore, in the future, vital registrations systems will need to be especially careful to ensure proper attribution to the underlying cause of death among persons with HIV and AIDS. Attribution is especially complicated because prolonged exposure to antiretroviral drugs, particularly protease inhibitors, may themselves contribute to the development of diabetes and heart disease [16].

How then should one determine the sequential order of the causes of death in a decedent who had been treated with ARVs and who also died with diabetes? As shown above, diabetes is clearly under-reported, even in a vital registration system that is generally considered to be complete and accurate [14]. So it is not surprising that at the same time that HIV and AIDS experts are advocating for better reporting of HIV and AIDS on the death certificate, diabetes experts are also advocating for better reporting of diabetes on the death certificate. Clearly, rules for proper attribution need to be developed to address these complex situations.

To improve cause-of-death reporting in the future, we recommend the following:

1. Continue to use the recently implemented two-part report (one part for the family that does not list HIV and the other part to be used by the Health Statistics Unit) to reduce the tendency for physicians to omit an AIDS diagnosis to alleviate families' fear of exposure [5].

2. Provide physician-focused trainings at hospitals and medical schools, emphasizing the importance of accurate vital statistics for the country and providing detailed instructions on proper completion of death registration forms.

3. Institute quality assurance, such as employing an on-site person responsible for ensuring completion of the form within 24 hours of death.

4. Implement incentives for accurate and complete reporting of death registration forms and possibly implement consequences for inaccurate reporting.

5. Simplify the death reporting process by working closely with the physicians who complete the forms, creating standards, and possibly mandating these standards.

6. Identify staff at the Ministry of Health who, with proper training, are charged with the responsibility of ensuring quality of death registration forms on a quarterly or semi-annual basis.

7. Encourage the practice of data utilization for decision making.

8. Continue to expand HIV and AIDS testing so that the condition does not remain hidden.

In the short term, improvements in vital registration systems may cause some difficulty in interpreting mortality trends and attributing declines to programme successes. However, in the long term, accurate mortality statistics will provide the country with many benefits, including: the ability to monitor the impact of programmes that have been scaled up to the population level; the ability to compare mortality across districts, allowing studies of best prevention and treatment practices; and the opportunity to track the impact of emerging diseases, such as diabetes and obesity.

In conclusion, this study shows that HIV- and AIDS-related deaths are substantially under-reported in Botswana. However, it is clear that the government is committed to improving its vital registration system as part of its national strategy to significantly impact the HIV and AIDS epidemic by 2016 (Botswana's Vision 2016 goals). Periodic studies, such as the one reported here, will allow the country to monitor improvements in its vital registration system. The goal of a complete and effective system is expected to be accomplished in the near future.

## Competing interests

The authors declare that they have no competing interests.

## Authors' contributions

NT conceived the study, and participated in its design and coordination and helped draft the manuscript. JW provided a comprehensive analysis of the data, interpreted the results, and took a lead role in writing the manuscript. SB participated in its design and coordination, and helped draft the manuscript. LP participated in the analysis and interpretation of the data, and took a lead role in writing the methods and results of the study. DM participated in the study and coordinated study activities including access to the vital registration data. ES helped with interpretation of the data, and provided advice on the design of the study. TR helped to conceive the study, and participated in its design and coordination. GK provided administrative support during study implementation and helped in data analysis. E-H S participated in the design and coordination of the study.

## Appendix 1

### Methods for reclassifying the causes of death as probable or possible HIV- and AIDS-related*

We varied the definition of HIV- and AIDS-related deaths by asking independent clinicians, who are employed by the Centers for Disease Control and Prevention (CDC) and the University of California, San Francisco, as HIV and AIDS experts, to review the -consensus causes of deaths, to examine the individual items on the abstract forms, and to use their clinical judgment to render an expert opinion on whether the death was truly HIV- and AIDS-related. In doing so, the experts used three different algorithms that resulted in classifications of "definitively HIV- and AIDS-related", "probably HIV- and AIDS-related", or "possibly HIV- and AIDS-related".

"Definitively HIV- and AIDS-related" deaths were determined in the following manner. Experts classified records in which the consensus cause of death was "HIV", "AIDS", "AIDS-related complex", or any HIV-related opportunistic illnesses as definitely HIV- and AIDS-related deaths. If all of the consensus causes of death were missing (<1% of all deaths), a death was coded as HIV- and AIDS-related if one or more of the individual physician reviews indicated the conditions listed above.

"Probably HIV- and AIDS-related" deaths were determined by the experts examining records, in which the individual physicians' abstract forms noted that the decedent was diagnosed with any WHO Stage IV condition during hospitalization prior to death, had a positive HIV test result, was noted to be receiving HIV-specific care, on ART, treated for Kaposi's sarcoma or *pneumoncystis jiorvecii *pneumonia (PCP), had a viral load test result available, or receiving cotrimoxazole for PCP prophylaxis. The causes of death from these records were reviewed again and classified as *probably *HIV- and AIDS-related deaths if the causes contained conditions listed in Section A.

"Possibly HIV- and AIDS-related" deaths were determined as above, except that causes also included the conditions listed in Section B.

When experts used the "definitive" definition, we labelled this as Method B. When experts used the definitive or probably definition, we labelled this as Method C. Finally, we defined Method D as experts using the definite, probable or possible definition. These three methods provided us with a range of estimates, from a conservative definition of HIV- and AIDS-related death (Method B) to a very liberal definition of an HIV- and AIDS-related death (Method D).

Section A: Probable HIV- and AIDS-related deaths if the cause of death was:

• Abscess, brain or lung

• Acute encephalopathy

• Cervical cancer

• Lymphoma (including non-Hodgkin's)

• Cardiomyopathy

• Dehydration

• Dementia (if <60 year old)

• Diarrhea/gastroenteritis

• Disseminated intravascular coagulation

• Electrolyte imbalance

• Empyema

• Encephalitis

• Endocarditis

• Hepatic failure/hepatitis

• Mediastinal mass

• Meningitis

• Multi-organ failure

• Pancreatitis

• Pericardial effusion

• Peritonitis

• Pleural effusion

• Pneumonia

• Prematurity

• Respiratory failure (no other cause)

• Sepsis

• Tuberculosis (pulmonary and extrapulmonary).

Section B: Possible HIV- and AIDS-related deaths if the cause of death was:

• Adrenal insufficiency

• Ascites

• Bronchiectasis, pneumoconiosis, pulmonary fibrosis

• Congestive heart failure (if <45 years old)

• Pulmonary edema

• Renal failure

• Stevens Johnson syndrome

• Stroke.

*Only in the sample of records in which data from the chart abstraction form suggested that the decedent was HIV infected

## Supplementary Material

Additional file 1 **Morbidity, mortality, and obstetric in-patient form, Botswana, 2003**. This form is completed by physicians for all in-patients in health facilities and is used by the Ministry of Health to monitor deaths. Click here for file

## References

[B1] AbouZahr C Boerma T Health information systems: the foundations of public health Bull World Health Organ 2005 12 8 578 583 16184276 PMC2626318

[B2] Mathers CD Counting the dead and what they died from: an assessment of the global status of cause of death data Bull World Health Organ 2005 12 3 171 177 15798840 PMC2624200

[B3] Sibai AM Mortality certification and cause-of-death reporting in developing countries Bull World Health Organ 2004 12 2 83 15042227 PMC2585897

[B4] Byass P Who needs cause-of-death data? PLoS Med 2007 12 11 e333 doi:10.1371/journal.pmed.0040333. 10.1371/journal.pmed.0040333 18031198 PMC2082647

[B5] King MB AIDS on the death certificate: the final stigma BMJ 1989 12 6675 734 736 10.1136/bmj.298.6675.734 2496825 PMC1835976

[B6] Burger EH Merwe L van der Volmink J Errors in the completion of the death notification form SAMJ 2007 12 11 1077 1081 18250917

[B7] Central Statistics Office, Ministry of Health, Botswana Health Statistics Report 200410.1097/00002030-200311210-00014

[B8] World Health Organization The WHO Family of International Classifications Geneva: WHO http://www.who.int/health-topics/classification/en/

[B9] Doctor HV Weinreb AA Estimation of AIDS adult mortality by verbal autopsy in rural Malawi AIDS 2003 12 2509 2513 10.1097/00002030-200311210-00014 14600523

[B10] Lopman BA Barnabus RV Boerma JT Creating and validating an algorithm to measure AIDS mortality in the adult population using verbal autopsy PLoS Medicine 2006 12 e312 DOI:10.137/journal.pmed.0030312 10.1371/journal.pmed.0030312 16881730 PMC1526767

[B11] Groenewald P Nannan N Bourne D Laubscher R Bradshaw D Identifying deaths from AIDS in South Africa AIDS 2005 12 193 201 10.1097/00002030-200501280-00012 15668545

[B12] SAS Institute Inc SAS 9.1.3 Help and Documentation 2000 Cary, NC: SAS Institute Inc

[B13] Chandramohan D Setel P Quigley M Effect of misclassification of causes of death in verbal autopsy: can it be adjusted? International Journal of Epidemiology 2001 12 509 514 10.1093/ije/30.3.509 11416073

[B14] Cheng WS Wingard DL Kritz-Silverstein D Barrett-Connor E Sensitivity and Specificity of Death Certificates for Diabetes Diabetes Care 2008 12 2 279 284 10.2337/dc07-1327 17959866 PMC2654202

[B15] Letamo G Prevalence of and factors associated with HIV- and AIDS-related stigma and discriminatory attitudes in Botswana Journal of Health Population and Nutrition 2003 12 4 347 35710.1371/journal.pone.0001531 15038590

[B16] Pacheco AG Tuboi SH Faulhaber JC Harrison LH Schechter M Increase in non-AIDS related conditions as causes of death among HIV-infected individuals in the HAART era in Brazil PLoS ONE 2008 12 1 e1531 10.1371/journal.pone.0001531 18231611 PMC2211396

