# Supplementary figures and images for: Validation of AIDS-related mortality in Botswana
Source: J Int AIDS Soc. 2009 Oct 24;12:24. doi: 10.1186/1758-2652-12-24 (PMC2775019; doi:10.1186/1758-2652-12-24)

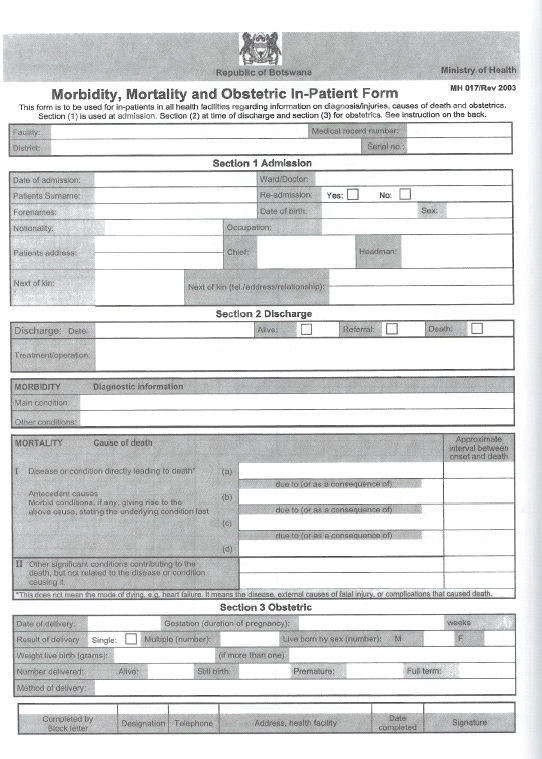

Supplement: Additional file 1 — Morbidity, mortality, and obstetric in-patient form, Botswana, 2003. This form is completed by physicians for all in-patients in health facilities and is used by the Ministry of Health to monitor deaths. [file 1758-2652-12-24-S1.DOC]
